# Characteristics of *Chlamydia suis* Ocular Infection in Pigs

**DOI:** 10.3390/pathogens10091103

**Published:** 2021-08-29

**Authors:** Christine Unterweger, Aleksandra Inic-Kanada, Sara Setudeh, Christian Knecht, Sophie Duerlinger, Melissa Stas, Daisy Vanrompay, Celien Kiekens, Romana Steinparzer, Wilhelm Gerner, Andrea Ladinig, Talin Barisani-Asenbauer

**Affiliations:** 1 University Clinic for Swine, Department for Farm Animals and Veterinary Public Health, University of Veterinary Medicine, 1210 Vienna, Austria; 1045238@students.vetmeduni.ac.at (S.S.); christian.knecht@vetmeduni.ac.at (C.K.); sophie.duerlinger@vetmeduni.ac.at (S.D.); melissa.stas@vetmeduni.ac.at (M.S.); andrea.ladinig@vetmeduni.ac.at (A.L.); 2 Institute of Specific Prophylaxis and Tropical Medicine, Center for Pathophysiology, Infectiology and Immunology, Medical University of Vienna, 1090 Vienna, Austria; aleksandra.inic-kanada@meduniwien.ac.at; 3 Laboratory for Immunology and Animal Biotechnology, Department of Animal Science and Aquatic Ecology, Faculty of Bioscience Engineering, Coupure Links, 654, 9000 Ghent, Belgium; Daisy.Vanrompay@UGent.be (D.V.); celien.kiekens@ugent.be (C.K.); 4 Institute for Veterinary Disease Control, Austrian Agency for Health and Food Safety (AGES), Robert Koch Gasse 17, 2340 Moedling, Austria; romana.steinparzer@ages.at; 5 Institute of Immunology, Department of Pathobiology, University of Veterinary Medicine, 1210 Vienna, Austria; wilhelm.gerner@vetmeduni.ac.at; 6 OCUVAC Centre of Ocular Inflammation and Infection, Laura Bassi Centre of Expertise, Center of Pathophysiology, Infectiology and Immunology, Medical University of Vienna, 1090 Vienna, Austria; talin.barisani@meduniwien.ac.at

**Keywords:** *Chlamydia suis*, piglets, experimental conjunctival infection, serology, antibodies, immunohistochemistry, lacrimal gland

## Abstract

*Chlamydia* (*C.*) *suis* can often be isolated from conjunctival swab specimens from pigs with conjunctivitis or keratoconjunctivitis. In the field, it is assumed to be a multifactorial disease triggered by immunosuppressing factors. This is the first experimental study to provoke clinical signs of conjunctivitis in pigs after *C. suis* primary mono-infection. Five six-week-old male piglets, free of ocular chlamydia shedding and seronegative for Chlamydia, were conjunctivally infected with the *C. suis*-type strain S45 (1 × 10^9^ inclusion forming units), while four piglets served as negative controls. The infection group developed clinical signs of conjunctivitis with a peak in the first week post-infection. Immunohistochemical evaluation revealed the presence of Chlamydia not only in the conjunctival epithelium, but also in the enlarged lacrimal glands, lungs, and intestine. No circulating antibodies could be detected during the whole study period of three weeks, although three different test systems were applied as follows: the complement fixation test, MOMP-based *Chlamydiaceae* ELISA, and PmpC-based *C. suis* ELISA. Meanwhile, high numbers of IFN-γ-producing lymphocytes within PBMC were seen after *C. suis* re-stimulation 14 days post-infection. Hence, these data suggest that entry via the eye may not elicit immunological responses comparable to other routes of chlamydial infections.

## 1. Introduction

Chlamydial infections are assumed to be widespread in commercial pig production and wild boars in Europe [1]. Chlamydia are obligate intracellular, Gram-negative bacteria causing a broad range of diseases in animals and humans. *Chlamydia* (*C.*) *suis* is considered the most prevalent chlamydial species in pigs [1,2], and its zoonotic potential has been proven [3,4,5]. In swine, *C. suis* infections have been primarily associated with asymptomatic or endemic subclinical infections [6], but also with a variety of clinical signs such as conjunctivitis [7,8], respiratory infections [9], enteritis [10,11], polyarthritis [12], and reproductive disorders [13,14,15,16,17]. In different European countries, seropositivity for *Chlamydia* spp. in pigs and wild boars differs enormously and ranges from around 30% to 96.5% [18,19,20,21]. Notably, serological tests showed varying results depending on the test system used. However, specific data on the seroprevalence of *C. suis* in domestic pigs at the country level, not within individual herds, hardly exist.

The transmission and pathogenesis of *C. suis* are far from being fully understood. Common infection sources, infection routes, vectors, and infection kinetics on pig farms are unknown. The bacteria is most often detected in the intestine [22,23] but not necessarily associated with clinical signs [18,23]. *C. suis* can also be regularly found in faeces [6,24,25]. Therefore, transmission via the faecal-oral route seems obvious [23], as well as transmission via aerosols or direct contact [8]. Chlamydia is isolated from the conjunctiva of both clinically healthy and symptomatic pigs [26,27]. After Chlamydia replicates in the conjunctival epithelial cells, conjunctivitis, often combined with seromucous discharge, can be observed, but not every conjunctival infection leads to clinical signs of conjunctivitis [7]. Chlamydial presence on the ocular mucosal surface potentially serves as a source for further spread [8]. Critical factors for the variation of ocular virulence seem to include the infectious dose, the number of repeated infections, age and immune status of the pig, possible co-infections, and genetic variations of the strains [26,28,29]. It is suspected that the intensification of pig production is responsible for the clinical manifestation of chlamydial ocular infection [8]. Due to intensive housing conditions for up to 15 weeks or longer in confined spaces, which is usually the case in pig fattening, *C. suis* reinfections can progress unrestricted and the infection pressure can increase, whereby sooner or later conjunctivitis will be recognizable in some but not all pigs due to the exposed locations of the eyes.

It is assumed that co-factors such as on-farm climate, dust, or immunosuppressive factors reinforce clinical signs. Conjunctivitis can be recognized early since the ocular surface is easily visible and more accessible to examination and sampling than the urogenital tract in sows; therefore, urogenital infection with *C. suis* is usually recognized at a late stage of inflammation [15].

Experimental data on ocular infections with *C. suis* are limited. Rogers and Anderson (1999) infected 12 three-day-old gnotobiotic piglets with the *C. suis* strain H7 [7]. While no conjunctival or other ocular clinical signs could be induced, diarrhoea could be seen from day seven post-infection (D7) in some infected animals, confirming the spreading of *C. suis* to other parts of the body. Nevertheless, typical *C. suis*-induced histopathologic lesions were recorded in conjunctival specimens and the gut. Immunohistochemical evaluations revealed chlamydial antigens in the conjunctival epithelium only on D7, but not on D14, D21, and D28. Chlamydial re-isolation from conjunctival mucosae was successful on D7 only.

Currently, no information on the production of antibodies after *C. suis* ocular infections is available. For practicing swine veterinarians, a plausible interpretation of positive or negative antibody results, which in routine diagnostics are measured by the complement fixation test (CFT) in most European countries, is of utter importance. Suboptimal specificity and especially cross-reactivity preclude its use for species-specific diagnosis and complicate the interpretation of results. However, sensitive and specific in-house ELISA-based tests would be another option to test pigs, since it is expected that chlamydial infections of the reproductive, intestinal, and respiratory tract trigger a specific humoral immune response.

Our study aimed to evaluate the distribution of *C. suis* S45 in organs besides conjunctiva after a primary experimental conjunctival infection and to measure levels of *C. suis*-specific antibodies using (i) the complement fixation test, (ii) an in-house *Chlamydiaceae*-specific major outer membrane protein (MOMP) ELISA [19,30], and (iii) a *C. suis*-specific polymorphic outer membrane protein C (PmpC)-based ELISA [31] in piglets over three weeks after infection. A potential recognition of *C. suis* antigens by T cells was investigated by IFN-γ ELISpot assays.

## 2. Results

### 2.1. Clinical Examination

**Figure 1:**
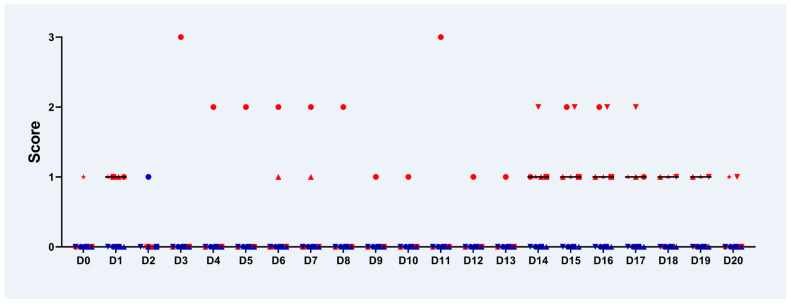
Individual faecal scores in *C. suis*-infected animals (n = 5, red symbols) and control animals (n = 4, blue symbols) starting on the day of infection until D20 based on the following score system: score 0: physiological; score 1: pasty; score 2: liquid with texture; score 3: watery without texture. Horizontal black bars show the median.

None of the infected or control piglets developed severe clinical signs at any time during the study. No obvious differences in average daily weight gain were seen between infected and control pigs (Figure S1). Two pigs of the infected group showed an inner body temperature higher than 40 °C on study day (D2), between D6 and D8, and additionally on D10. From D11 onwards, all infected piglets had physiological inner body temperatures (38.5 °C–39.5 °C). Control animals never developed a fever. Between D14 and D17, infected piglets showed pasty to liquid faeces, while faeces of control piglets had a normal consistency (Figure 1). Increased salivation in all *C. suis*-infected animals starting from D5 was recorded.

### 2.2. Examination of the Eyes

**Figure 2:**
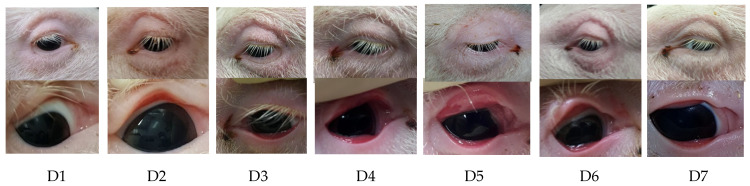
Swelling of the upper and lower eyelids, reddening of the conjunctiva, and ocular discharge in *C. suis*-infected piglets. One eye from one representative infected piglet is shown. Images placed on top of each other belonged to the same animal, in the upper row as an overview image and in the lower row as a close-up image.

Left and right eyelids from all infected piglets and tarsal conjunctiva showed moderate to severe reddening starting on D2 post-infection; the reddening was observed over several days and continuously declined before disappearing completely on D10. Upper and lower eyelids were oedematous between D2 and D6 as well as from D12 until termination (D21) (Figure 2).

**Figure 3:**
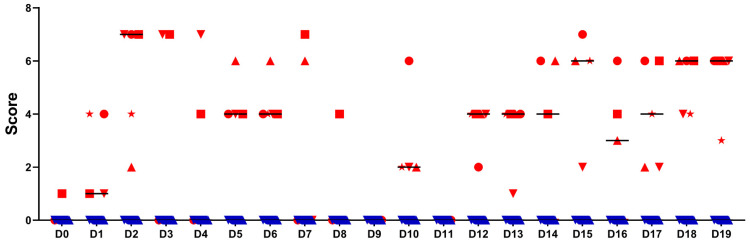
Sum of individual eye scores based on the assessment of reddening of the bulbar and tarsal upper and lower conjunctiva, oedema of the upper and lower eyelid, and ocular discharge in the infected group (n = 5, red individual symbols) and the control group (n = 4, blue individual symbols) on 20 consecutive days. Horizontal bars show the median. The highest possible score per animal per day was 9.

During the first occurrence, eyelid oedema was scored as moderate to severe, while during the second occurrence, eyelid oedema was mild. The sum of scores from reddening of the tarsal and bulbar upper and lower conjunctiva and oedema of the upper and lower eyelids is presented in Figure 3. While the control animals did not show any clinical signs, *C. suis*-infected animals showed clear clinical signs, particularly on D2 and D3. Notably, alterations were noticeable in both eyes of most piglets, although only the right eye was infected.

Ocular serous discharge was recorded at three periods: between D1 and D3 (low degree), on D10 (high degree), and between D14 and D21 (high degree). In the last study week, ocular discharge was combined with continuous serous nasal discharge in 3/5 piglets. Follicles and corneal abnormalities were not recorded at any time.

### 2.3. Ocular Shedding

**Figure 4:**
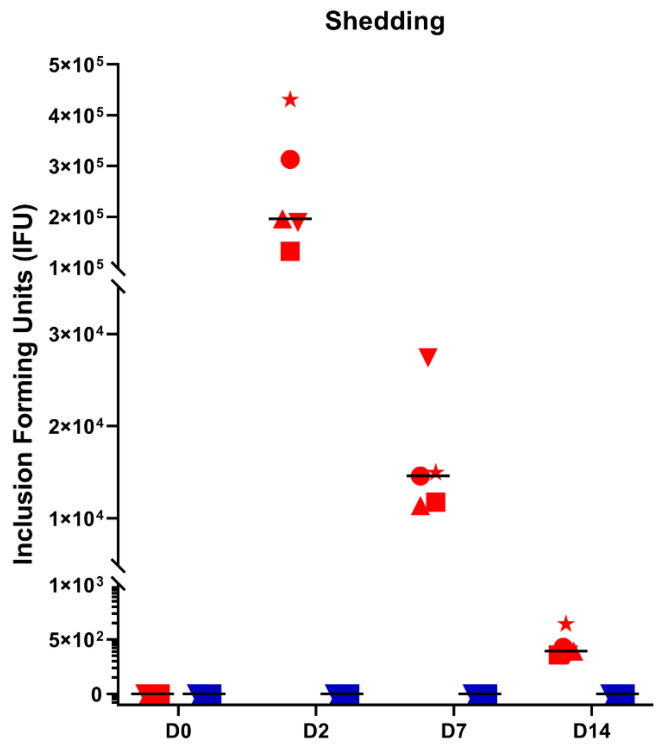
*C. suis* inclusion forming units (IFU) found in conjunctival swabs by isolation on McCoy cells on D0, D2, D7, and D14 in infected (red symbols) and control (blue symbols) animals. High shedding was observed two days after infection but with a high animal to animal variation. No shedding was seen in the controls. Shedding lasted at least until D14.

Right before the infection with *C. suis*, animals did not shed any Chlamydia. Ocular *C. suis* shedding was at its peak two days after infection. Still, it differed from one individual to another. In the infected group, 14 days after infection, *C. suis* shedding decreased to low numbers (<50.000 IFU) and was not seen any longer on D21 (Figure 4). In the control group, no *C. suis* were detected in the eyes of any animal at any time point.

### 2.4. Serology

No *C. suis*-specific antibodies could be measured using either the CFT, the MOMP-based ELISA, or the PmpC-based ELISA in all piglets at the time of infection. In addition, no seroconversion could be seen by those three assays at any time during the three-week infection trial, neither in the infection group nor in the control group.

### 2.5. IFN-γ Production in Blood-Derived Lymphocytes

**Figure 5:**
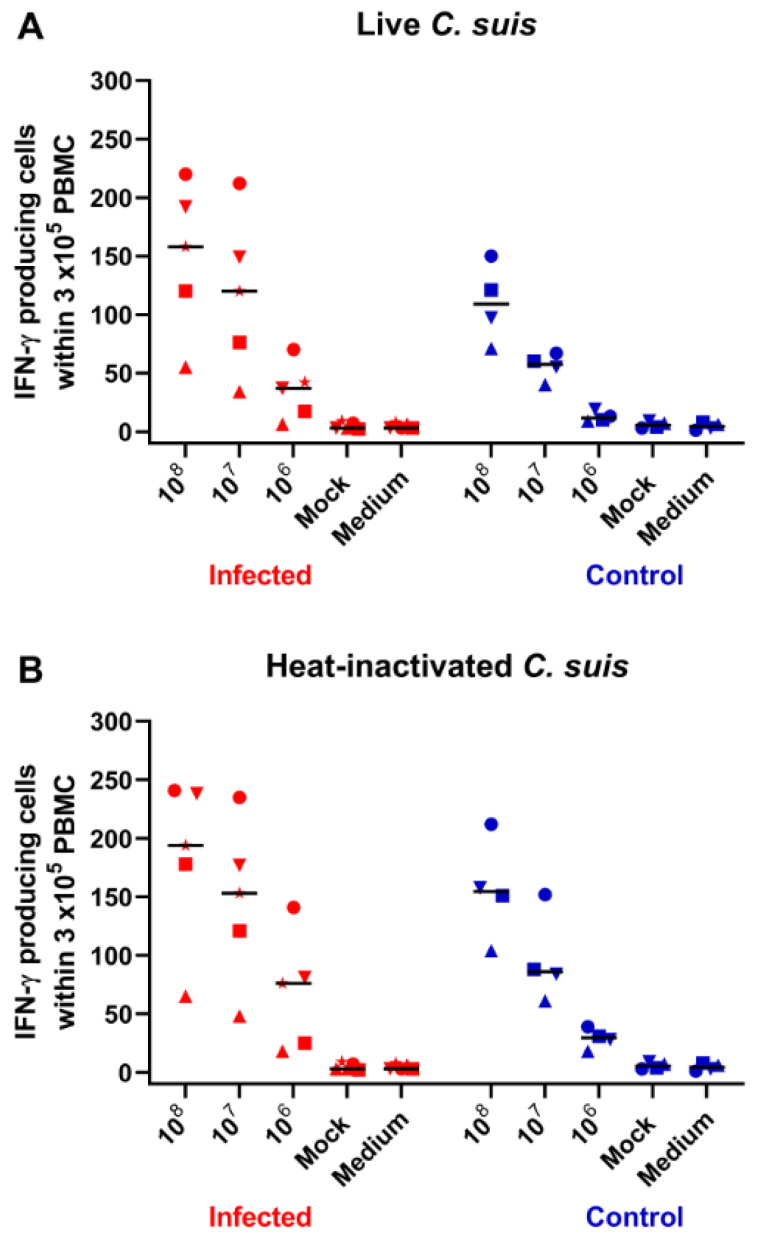
(**A**,**B**) IFN-γ-producing cells in blood following *C. suis* antigen re-stimulation in vitro. PBMC isolated on D14 were re-stimulated with different doses of live and heat-inactivated *C. suis* antigens, and IFN-γ production was investigated by ELISpot. Each symbol represents the number of IFN-γ-producing cells within 3 × 10^5^ PBMC for an individual animal, representing the mean of triplicate microcultures. Results for infected animals are shown in red, and for control animals in blue symbols. Cells cultivated in medium or mock supernatants served as negative controls. Numbers on the X-axis give the number of inclusion forming units (IFU) per mL, determined prior to heat inactivation. Horizontal bars show the median.

To identify a potential recognition of *C. suis* antigens by circulating T cells, IFN-γ ELISpot assays were performed. PBMC were isolated at D14 and re-stimulated with live or heat-inactivated *C. suis*. The number of IFN-γ-producing cells was dose dependent, regardless of live or heat-inactivated *C. suis* preparations being used for re-stimulation (Figure 5A, B, respectively). Additionally, IFN-γ-producing cells in *C. suis*-stimulated microcultures were clearly above medium and mock-stimulated PBMC. However, in PBMC isolated from control pigs, the numbers of IFN-γ-producing cells were nearly as high as in infected pigs, with only marginally increased spot counts between infected and control pigs for the same amount of *C. suis* antigens.

### 2.6. Pathomorphological Findings

Macroscopic lesions were absent in all pigs of the control group. Three weeks after infection, no macroscopic lesions could be seen in the eyes of the infected piglets. Still, a remarkable infection detected by immunohistochemical staining and inflammation of the lacrimal glands from all five piglets, which were twice to three times as large as those of the control animals, was recorded.

### 2.7. Immunochemistry

**Figure 6:**
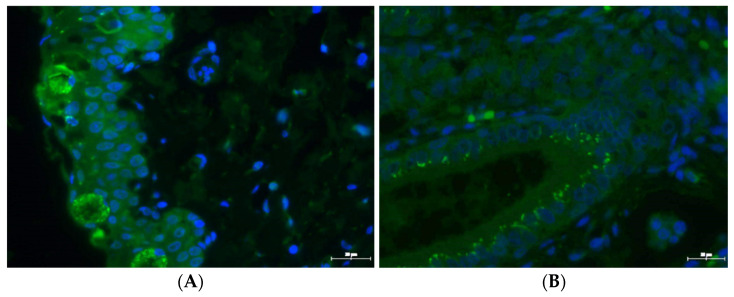
IFU in the conjunctiva (**A**) and glandula lacrimalis (**B**); representative images are shown. *C. suis* inclusions are identified using an FITC-labelled anti-*Chlamydia* LPS antibody (green dots). Cells were counterstained with DAPI (blue). Original magnification was 40×. bar = 20 μm.

*C. suis* inclusions in diverse tissues (conjunctiva, lacrimal glands, duodenum, and lungs) were detected on the day of necropsy (D21) in the infected group (Figure 6A,B). Especially goblet cells appeared to harbour *C. suis.* Three animals of the control group also had positive staining results in the duodenum, and control animal 3 additionally showed single inclusions in conjunctiva and glandula lacrimalis. *C. suis* inclusions were not observed in the kidneys and testes (Table 1). 

## 3. Discussion

In this study, we investigated the characteristics of the conjunctival *C. suis* infection in pigs. It is known that *C. suis* can be isolated from conjunctival swabs of pigs with conjunctivitis or keratoconjunctivitis, but at the same time also from asymptomatic pigs [8,26]. The conjunctival Chlamydia prevalence of asymptomatic pigs in intensive housing is much higher than in pigs from extensive housing [8]. Co-infections and unfavourable environmental influences, such as poor stable climate, draughts, or overcrowding, can be predisposing factors for clinically visible ocular *C. suis* infections [8]. Especially overcrowding increases the risk of ocular chlamydial contact after direct exposure to the droplets expelled by shedding individuals in close contact. The area exposed to aerosols is much more prominent on the ocular surface when compared to the mouth and nose [32]. The eye might have a crucial role in chlamydial transmission [8], as it is assumed in the course of human and animal respiratory viral diseases [33].

In the present study, co-infections except for usual early colonizers in pigs and environmental triggers could nearly be excluded. No clinical signs were seen during the study in the control pigs, except for one piglet that had to be euthanized at a very early time of the trial prior to D0 (day of infection) due to severe streptococcal arthritis and periarthritis with no likelihood of recovery without the use of antibiotics. At the same time, piglets of the infected group developed clinical signs, such as conjunctivitis, swelling of the eyelids, and ocular discharge with varying degrees, especially during the first seven days post-infection. Therefore, this is the first experimental study to show that ocular *C. suis* infection in conventional pigs leads to clinical signs. The only other ocular *C. suis* infection trial by Rogers and Anderson (1999) was performed in gnotobiotic animals that showed only asymptomatic conjunctivitis [7]. The two studies also showed apparent differences in the length of ocular shedding after infection—at least 7 days in the older study and at least 14 days in the present study—and the immunohistochemical presentation of the chlamydial antigen. Rogers and Anderson (1999) could not detect Chlamydia in conjunctival specimens at any time point later than seven days after the infection, but was shown to be present in conjunctival specimens for at least three weeks in the current study.

In principle, the comparison of data from a trial with gnotobiots and those from conventional nursery or growing piglets is critical; no other bacteria besides the chlamydial infection isolate are present in gnotobiots, and Chlamydia can spread much easier and faster than in older piglets, which have a completely different microbiome. Apart from this, scientific methods used in the 1990s are also not comparable with the current state of the art. Additionally, both studies differed in the infection isolate, infection dose, and housing conditions. 

The assumption that the shedding of Chlamydia on D2 is due to recovery from instilled Chlamydia is open to question. However, topically instilled substances are rapidly cleared from the ocular surface within seconds through blinking and tear turnover, and in humans, the tear film is restored every 2–3 min [34]. Therefore, it is unlikely that the results reflect Chlamydia residuals, but rather show the dynamics of active infection paralleling dynamics described for *C. suis* infection in vitro. The intracellular cycle of *C. suis* takes about 48 h [35], which could explain the chlamydial shedding after two days. This short shedding period is in line with Rogers and Anderson (1999), but in other infection trials in sows with *C. suis* S45 where a different (vaginal) route of infection was chosen, vaginal excretion was still present at day 56 post-infection [36].

Ocular clinical signs disappeared on D10, and eyelid oedema reoccurred in some animals between D13 and the end of the trial, indicating a *C. suis* reinfection. Pig veterinarians usually classify eyelid oedema as oedema disease, a very common *E. coli*-induced disease primarily seen after weaning. However, as the manifestations of eyelid oedema observed in this study are identical to those of oedema disease, one should include chlamydial infection as a differential diagnosis.

For the first time, we were able to show that *C. suis* can colonize the lacrimal glands. In infected animals, the lacrimal glands were also at least three times larger than in control animals. The chlamydial antigen could not be detected in the cornea, which is in line with the results by Rogers and Andersen (1999). Thus, we can assume that Chlamydia actively enters the lacrimal gland against the tear flow, whereas with the tear flow, they are indeed drained down the tear duct reaching the nasopharynx, from where they can be either inhaled or swallowed.

After 21 days, *C. suis* was detected in the lungs of one animal. It would be interesting in future experiments to test whether, after repeated infections over a longer time, more animals would test positive in lungs and other compartments. The increased salivation in all *C. suis*-infected animals over the entire period of the study was also striking, which has never been described before in connection with chlamydial infections in pigs. Unfortunately, the salivary glands were not examined histologically and, therefore, only assumptions of a connection to chlamydial infection can be made.

Immunohistochemistry data confirmed a *C. suis* infection in the duodenum at necropsy time, not only in the infected group, but also in the control group. The rectal shedding was not analysed at any time point, which is undoubtedly a weakness of this study. Despite the presence of inclusions in the duodenum of controls, no alterations in faecal consistency were present. It can be assumed that the intestinal tract of the piglets had already been colonized with *C. suis* before the beginning of the trial, which is not unusual for conventionally reared piglets that were weaned some weeks earlier, as we can confirm from our own routine experience. Since clinical signs are most likely dependent on the infection dose, this could explain the absence of clinical symptoms in the control group. It clearly shows that even in colonized piglets, a superinfection may lead to clinical signs, at least infections of the eyes. We doubt systemic spreading via blood since no Chlamydia was detected in the kidneys and testes of infected animals, at least during the investigated study period.

Our results from the IFN-γ ELISpot might also confirm that the pigs encountered field-circulating *C. suis* before the performed experimental infection. This speculation is based on the observation that the numbers of IFN-γ-producing lymphocytes identified in the control groups were close to the numbers found in the infected group. If this holds, the experimental infection performed in this study would have caused only a slight additional increase in the number of blood-circulating *C. suis*-specific IFN-γ-producing lymphocytes. Another explanation for the unexpectedly high number of IFN-γ-producing lymphocytes within the PBMC of the control piglets could be the stimulation of the cells with a whole *C. suis* antigen. This may have contained conserved bacterial components that stimulate cells of the innate immune system present within PBMC, which in turn may have the capacity to stimulate non-*C. suis*-specific T cells for a bystander or non-cognate production of IFN-γ, as described for *Salmonella*, a pathogen that partially also resides inside of cells [37]. Purified recombinant *C. suis* proteins may provide a possibility to circumvent this phenomenon. Indeed, in a study conducted in *C. trachomatis* seropositive versus seronegative women, higher numbers of IFN-γ-producing cells were found in PBMC of seropositive individuals following in vitro stimulation with recombinant PmpF and MOMP [38]. However, gradient-purified elementary bodies also caused such differences. Hence, these results suggest that a comprehensive antigenic toolbox for *C. suis* in vitro stimulation may allow for the use of IFN-γ ELISpots as an additional assay to study the immune response against Chlamydia in pigs in future experiments.

Despite the findings of a chlamydial antigen in a series of specimens and the suspicion of a previous infection or at least colonization of the gut prior to infection, no antibodies could be measured during the whole study period of three weeks. Serological testing using CFT is a widely used antibody detection method. In most countries, ELISA-based tests are not commercially available for routine diagnostics. Szeredi et al. (1996) could show that positive ELISA results and CFT titres showed poor agreement [18]. The CFT is also known to have poor sensitivity. This was our first explanation when we realized that, despite proven *C. suis* infection in the upper and lower respiratory and intestinal tract, no antibodies were detected. Even with two more sensitive and specific ELISAs, no antibodies could be measured at any time point within the study period of three weeks. Besides considering that only antibodies can be measured after a systemic disease, wrong time points of serum sampling could be discussed: it could be hypothesized that it needs more reinfections for immunoglobulin (Ig) G production. In the personal experience of routine diagnostics, we rarely observe positive CFT results, even if serology is only conducted in sows on a routine basis. Growing and fattening pigs are seldom tested for anti-chlamydial antibodies in routine diagnostics. Therefore, no data about antibody prevalence in growing pigs in the field exist. According to Den Hartog et al. (2006), negative serology in hospitalized psittacosis patients is not uncommon. They argue that Chlamydia serology can be negatively influenced by antibiotic use and genetic variations in some receptors, leading to inadequate recognition of Chlamydia by the host immune system. Antibiotic treatment in *C. suis*-induced conjunctivitis as a single finding is uneconomical and usually not carried out due to the associated compliance with the withdrawal time. Lack of seroconversion in a human case of psittacosis has also been described recently [39]. The route of infection might play a crucial role, since genital experimental *C. suis* S45 infections in sows led to an evident seroconversion, detected by an ELISA using purified S45 elementary bodies (EBs) as the antigen [36]: *C. suis* S45-specific serum IgM and IgG were observed from seven days post-primary infection onwards, and the mean titres peaked at 14 or 21 days post-infection. *C. suis* reinfection, which would reflect the situation of the current study, induced even higher IgM and IgG titres. Further investigations are necessary to learn more about the interpretation of seronegative animals and to figure out whether the animals are truly IgM and IgG negative, or if the test systems used are inappropriate, at least in the case of chlamydial ocular and intestinal infections.

## 4. Materials and Methods

### 4.1. C. suis

*C. suis* strain S45, kindly provided by Nicole Borel, University of Zurich UZH, was used to infect piglets. This strain was isolated in the late 1960s from the faeces of an asymptomatic Austrian pig [40] and is considered the type strain [1]. *C. suis* S45 was successfully used in several experimental studies in pigs before [10,41,42], but never in ocular infection trials.

### 4.2. Infection Experiment

Ten four-week-old conventionally raised male piglets (large white x landrace x pietrain), were brought into the animal biosafety level 2 facilities of the University Clinic for Swine, Vetmeduni, Vienna after weaning. The animals were housed in isolation units and fed ad libitum with a commercial starting diet without the addition of antibiotics. They were randomly divided into two groups of five pigs (control group A and challenge group B) and housed in separate compartments. They had permanent access to fresh water and enrichment material according to the Austrian law. Antibodies against Chlamydia were absent as determined by three different serological test systems: a *C. suis* PmpC-based ELISA, a MOMP-based ELISA, and the complement fixation test (CFT) using *C. abortus* as an antigen. Animals were free of Porcine Reproductive and Respiratory Syndrome Virus (PRRSV), Influenza virus A, and in all Austrian notifiable diseases. Ocular swabs were collected from each piglet prior to experimental inoculation and did not contain Chlamydia as determined by PCR and culture. Starting 7 days prior to infection (D-7), a daily clinical observation was performed that focused on both eyes including the (i) lower and upper eyelids, (ii) tarsal and (iii) bulbar conjunctiva, (iv) cornea, and (v) ocular discharge. A scoring for reddening and oedema/swelling of eyelids and reddening of conjunctiva, as well as for the cloudiness of cornea and presence of follicles and ocular discharge, was assigned. One single point was awarded if one or both eyes were affected. Therefore, an animal could receive points between zero and nine per day. The quality of ocular discharge was documented (serous, mucous, seromucous, and purulent). Additionally, general health including feed intake, rectal temperature, faecal scoring (physiological faeces was scored 0, pasty faeces was scored 1, liquid faeces with texture was scored 2, and watery faeces without texture was scored 3), and nasal discharge were recorded.

At the age of six weeks (D0), the challenge group was ocularly infected with the *C. suis*-type strain S45 by instillation of the inoculum (1 × 10^9^ inclusion forming units/animal, total volume 100 µl) in the right ventral conjunctival sac using a sterile pipette. The control group was inoculated with phosphate-buffered saline (PBS). Body weights were recorded individually at D-7, D0, D7, D14, and D21 (day of necropsy) and the average daily weight gain was calculated. For the quantification of *C. suis* EBs, conjunctival swab samples were collected from the right eye of all pigs on D-7, D0, D2, D7, and D14. Darcon swabs were used to swab the conjunctiva and placed in Copan Universal Transport Medium (UTM-RT) (Copan, Italy). Swabs were stored at −80 °C until chlamydial isolation. Blood samples from the jugular vein were collected for antibody detection on D-7, D2, D7, D14, and D21 and centrifuged (10,000× *g*, 10 min) for serum collection; heparinized blood samples from D14 were used for PBMC isolation. Sera were stored at −20 °C until further testing (Table 2).

All piglets from both groups were euthanized for necropsy on D21 by intravenous injection of a combination of ketamine hydrochloride (Narketan^®^ 10 ad us. vet., Vetoquinol Österreich GmbH, Vienna, Austria) and azaperone (Stresnil^®^ ad us. vet., Elanco GmbH, Cuxhaven, Germany), followed by intracardial injection of T61^®^ (Intervet GesmbH, Vienna, Austria). At necropsy, pigs were examined for gross lesions. Specimens from both eyes, including cornea, palpebral conjunctiva, and lacrimal glands of every pig, as well as specimens of lung, duodenum, ileum, colon, and testes, were selected for histologic staining methods. In the case of macroscopic pathological abnormalities, other organs were sampled, like kidneys of three pigs in the challenge group, which had a pale colour. Samples embedded in paraffin were used for immunohistochemical stainings. 

This study was approved by the institutional ethics and animal welfare committee and the Austrian national authority according to §§ 26ff. of Animal Experiments Act. Tierversuchsgesetz 2012—TVG 2012 (GZ68.205/0183-WF/V/3b/2017). 

### 4.3. Determination of Chlamydial Inclusion Forming Units (IFUs) from Conjunctival Swabs

IFUs were determined by inoculation of the obtained swab material onto confluent cultures of McCoy cells (ATCC^®^ CRL-1696™). Centrifugation at 200× *g* for 1 h was carried out to ensure attachment of EBs. After incubation for 24 h at 37 °C/5% CO_2_ and 95% humidity in the presence of 1mg/mL cyclohexamide (Sigma Aldrich, Steinheim, Germany), cells were fixed in ice-cold methanol and stained with a FITC-conjugated monoclonal antibody against Chlamydia LPS (1:20 in PBS, Clone B410F, Pierce Biotechnology, Rockford IL, USA). IFUs were recorded using an epifluorescence microscope (Zeiss AxioObserver, Zeiss, Jena Germany).

### 4.4. Immunohistochemistry

For immunohistochemical analyses, sections were deparaffinised and mounted on glass slides. For antigen retrieval, sections were treated with 1 mg/mL trypsin (Sigma Aldrich, St. Louis, MO, USA) in PBS for 10 min at 37 °C and blocked with 10% BSA (Sigma Aldrich, St. Louis, MO, USA) in PBS before incubation with a FITC-conjugated monoclonal antibody against Chlamydia LPS (1:20 in PBS, Clone B410F, Pierce Biotechnology, Rockford IL, USA) was carried out. A fluorescence microscope (Axio-Observer, Zeiss, Vienna, Austria) was used to verify the presence of IFU. Image acquisition was carried out using TissueFAXS software, v.6 (TissueGnostics, Vienna, Austria).

### 4.5. Antibody Responses

Sera were analysed using different methods for antibody detection: CFT [43], an in-house recombinant MOMP ELISA [19,30], and a PmpC-based ELISA [31]. Full-length recombinant MOMP of *C. suis* S45 was produced in COS-7 cells as previously described [44] and used to coat ELISA plates. The MOMP ELISA was performed as described by Kieckens et al. (2018), testing all sera first at a fixed dilution of 1/50. All positive samples were further titrated using 2-fold dilutions. MOMP ELISA results were also confirmed using a *C. suis*-specific antibody ELISA based on the use of a B cell epitope of the *C. suis* PmpC. The peptide representing the B-cell epitope (SSQQSSIAS) was synthetized, pHPLC purified, and analysed by MS-UPLC. The peptide contained an N-terminal acetyl group and was C-terminal, attached to polyethylene pins via incorporation of an extra cysteine. The peptide-coated pins were assembled on a 96-well polyethylene carrier (pin peptide ELISA format) for use. The PmpC ELISA was performed as described by De Puysseleyr et al. (2018), testing all sera first at a fixed dilution of 1/50. If positive, samples were titrated using 2-fold dilutions.

### 4.6. IFN-γ ELISpot from PBMC

PBMC were isolated from heparinized blood samples from D14 by density gradient centrifugation using Pancoll human, density 1.077 g/mL (PAN-Biotech, Aidenbach, Germany; 30 min at 920× *g*). After counting, 3 × 10^5^ PBMC/well were seeded in 96-well ELISpot plates (Merck-Millipore, Burlington, MA, USA) that were coated with anti-porcine IFN-gamma mAbs (clone plFNγ-I, Mabtech AB, Nacka Strand, Sweden). PBMC were re-stimulated with different doses of live or heat-inactivated *C. suis* preparations, ranging from 10^8^ to 10^6^ IFU per mL. PBMC cultivated in a cell culture medium (RPMI 1640 [PAN-Biotech] supplemented with 10% heat-inactivated foetal calf serum [Sigma, Schnelldorf, Germany], 100 IU/mL penicillin and 0.1 mg/mL streptomycin [PAN-Biotech]) served as the negative control. In addition, PBMC were cultivated with “mock” supernatants as an additional negative control. Here, the medium was added, which was used for the propagation of *C. suis* on their Caco-2 target cells, but the medium was derived from non-infected cells. The volume used of this medium was equivalent to the volume of the highest number of *C. suis* cells used for stimulation (i.e. 10^8^ IFU/ml). Plates were incubated for 20 h at 37 °C and 5% CO_2_. Thereafter, plates were washed five times and incubated with biontinylated anti-porcine IFN-gamma mAbs (clone P2C11, Mabtech). After further washing, a second incubation was performed with streptavidin-AP (Sigma). Spots were then visualized by the addition of 5-bromo-4-chloro-3-indolyl phosphate/nitro blue tetrazolium substrate (Sigma). After intense washing and drying of plates, spots were analysed and counted with an AID ELISpot reader (AID, Straßberg, Germany). 

### 4.7. Statistical Analysis

Due to the low number of animals included in the study and the pilot character, the results are only presented descriptively. Figures were created using GraphPad prism version 9.0.0 (GraphPad Software Inc., San Diego, CA, USA). In each graph, the result of each individual animal is shown, and the error bars represent the median. For the IFN-γ ELISPOT assay, each condition was performed in triplicates, for which the average was calculated with Microsoft Excel. Thus, the graphical representation depicts the calculated average for each individual animal per condition, and the error bar represents the median for each treatment group.

## 5. Conclusions

Porcine ocular chlamydial infections are hardly understood at least in terms of T-cell- and B-cell-based immune response, antibody production, pathogenesis, and clinical manifestation. It is not known whether ocular chlamydial infections induce measurable antibodies within a short period after infection or whether the current methods were simply not able to detect antibodies. The eye might serve as a chlamydial reservoir and more importance should be given to this fact in order to better understand epidemiological and transmission processes in the context of chlamydiosis.

## Figures and Tables

**Table 1:** Occurrence of *C. suis* inclusions in the organs of pigs experimentally exposed to *C. suis* and non-infected controls, evaluated by immunofluorescent staining methods.

Animal Number	Conjunctiva	Glandula Lacrimalis	Lungs	Duodenum	Kidney	Testes
control 1	−	−	−	−	−	−
control 2	−	−	−	+	−	−
control 3	(+)	(+)	−	+	−	−
control 4	−	−	−	+	−	−
*C. suis* 1	+	+	−	+	−	−
*C. suis* 2	+	+	−	+	−	−
*C. suis* 3	+	+	−	+	−	−
*C. suis* 4	+	+	+	+	−	−
*C. suis* 5	+	+	−	+	−	−

+: *C. suis*-positive cells detectable, (+): single inclusions visible −: no detectable *C. suis*-positive cells.

**Table 2:** Study design. W: weighing. CS: conjunctival swabs. S: serum. PBMC: peripheral blood mononuclear cells. Starting from D0, a daily clinical examination with special focus on the eyes was performed.

Animal Number	D-7	D0 Infection	D2	D7	D14	D21 Necropsy
control 1	W, CS	W, CS, S	W, CS, S	W, CS, S	W, CS, S, PBMC	W, CS, S
control 2	W, CS					
control 3	W, CS	W, CS, S	W, CS, S	W, CS, S	W, CS, S, PBMC	W, CS, S
control 4	W, CS	W, CS, S	W, CS, S	W, CS, S	W, CS, S, PBMC	W, CS, S
control 5	W, CS	W, CS, S	W, CS, S	W, CS, S	W, CS, S, PBMC	W, CS, S
*C. suis* 1	W, CS	W, CS, S	W, CS, S	W, CS, S	W, CS, S, PBMC	W, CS, S
*C. suis* 2	W, CS	W, CS, S	W, CS, S	W, CS, S	W, CS, S, PBMC	W, CS, S
*C. suis* 3	W, CS	W, CS, S	W, CS, S	W, CS, S	W, CS, S, PBMC	W, CS, S
*C. suis* 4	W, CS	W, CS, S	W, CS, S	W, CS, S	W, CS, S, PBMC	W, CS, S
*C. suis* 5	W, CS	W, CS, S	W, CS, S	W, CS, S	W, CS, S, PBMC	W, CS, S

## Data Availability

Not applicable.

## Supplementary Materials

The following are available online at https://www.mdpi.com/article/10.3390/pathogens10091103/s1, Figure S1: Body weight (kg) on individual days (D-7, D0, D7, D14, and D21) in control animals (blue) and *C. suis*-infected animals (red).
