# Supplementary figures and images for: Characteristics of Chlamydia suis Ocular Infection in Pigs
Source: Pathogens. 2021 Aug 29;10(9):1103. doi: 10.3390/pathogens10091103 (PMC8470092; doi:10.3390/pathogens10091103)

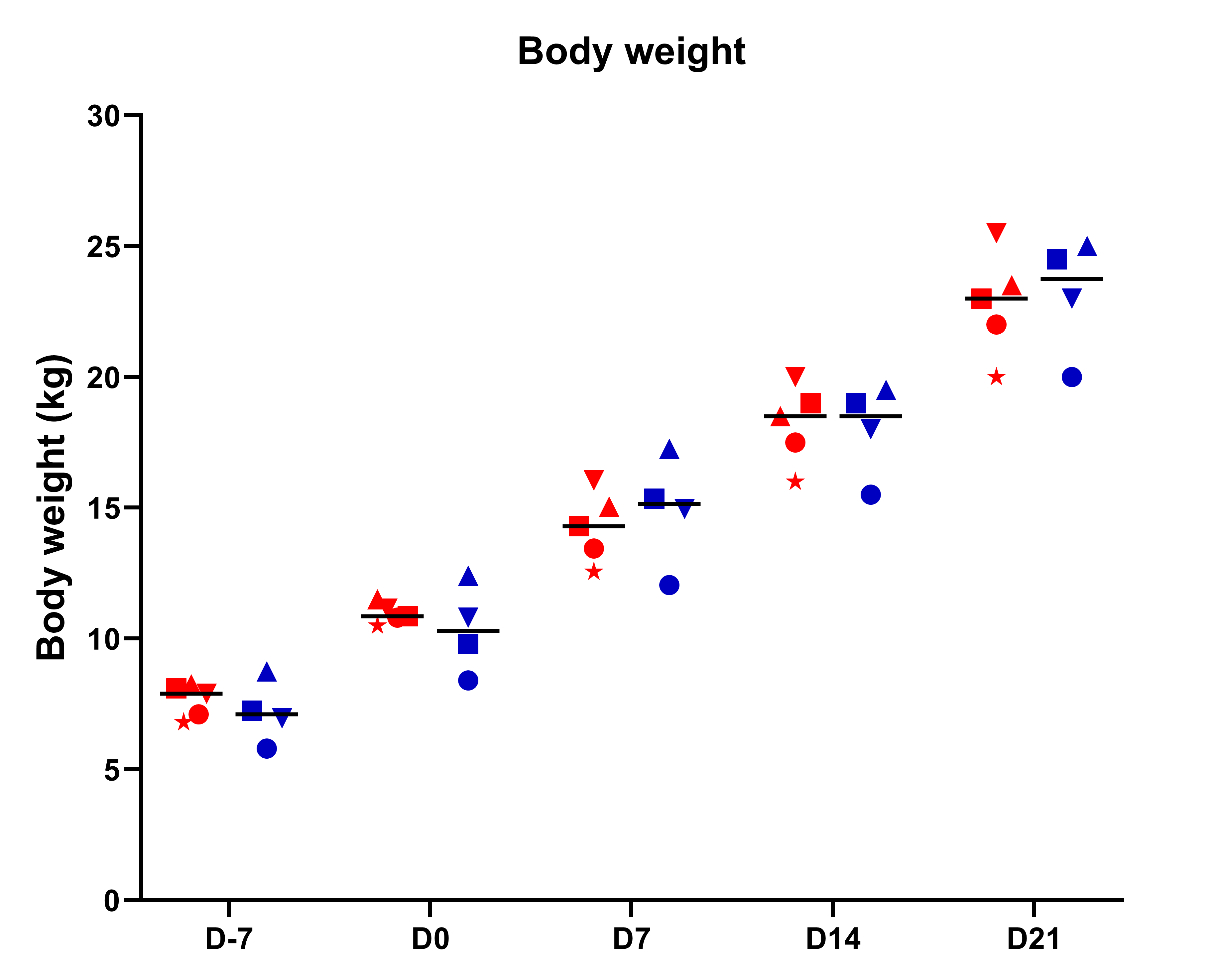

Supplement: Supplementary file 1 [file pathogens-10-01103-s001.zip › pathogens-1355329-supplementary.tif]
